# Diffusion and Interface Effects during Preparation of All-Solid Microstructured Fibers

**DOI:** 10.3390/ma7096879

**Published:** 2014-09-25

**Authors:** Kobelke Jens, Bierlich Jörg, Wondraczek Katrin, Aichele Claudia, Pan Zhiwen, Unger Sonja, Schuster Kay, Bartelt Hartmut

**Affiliations:** Leibniz Institute of Photonic Technology Jena, Albert-Einstein-Str. 9, D-07745 Jena, Germany; E-Mails: jens.kobelke@ipht-jena.de (K.J.); joerg.bierlich@ipht-jena.de (B.J.); katrin.wondraczek@ipht-jena.de (W.K.); claudia.aichele@ipht-jena.de (A.C.); zhiwen.pan@ipht-jena.de (P.Z.); sonja.unger@ipht-jena.de (U.S.); hartmut.bartelt@ipht-jena.de (B.H.)

**Keywords:** microstructured fiber, photonic crystal fiber, fiber manufacturing

## Abstract

All-solid microstructured optical fibers (MOF) allow the realization of very flexible optical waveguide designs. They are prepared by stacking of doped silica rods or canes in complex arrangements. Typical dopants in silica matrices are germanium and phosphorus to increase the refractive index (RI), or boron and fluorine to decrease the RI. However, the direct interface contact of stacking elements often causes interrelated chemical reactions or evaporation during thermal processing. The obtained fiber structures after the final drawing step thus tend to deviate from the targeted structure risking degrading their favored optical functionality. Dopant profiles and design parameters (e.g., the RI homogeneity of the cladding) are controlled by the combination of diffusion and equilibrium conditions of evaporation reactions. We show simulation results of diffusion and thermal dissociation in germanium and fluorine doped silica rod arrangements according to the monitored geometrical disturbances in stretched canes or drawn fibers. The paper indicates geometrical limits of dopant structures in sub-µm-level depending on the dopant concentration and the thermal conditions during the drawing process. The presented results thus enable an optimized planning of the preform parameters avoiding unwanted alterations in dopant concentration profiles or in design parameters encountered during the drawing process.

## 1. Introduction

For about two decades, microstructured optical fibers (MOF) have been intensively investigated due to their unique optical properties (e.g., unusual dispersion, endlessly single mode transmission, photonic band gap propagation) [1,2,3,4]. All-solid MOF, as a specific group of MOF show a few outstanding advantages compared to holey MOF. Due to their compact design they are robust and show a tensile strength behavior similar to standard silica fibers. The preparation approach typically follows the stack-and-draw method by arranging different doped rods or canes enclosed by a jacketing tube. The interstitial volume between the single packing elements is removed at least in the final drawing procedure by applying vacuum at moderate drawing temperatures, or at atmospheric pressure by operating with the effect of surface tension at higher drawing temperatures. While in case of stacks of undoped silica rods the simultaneous sintering and stretching faces no problems in terms of bubble formation or geometrical deformation, the use of doped silica rods or canes induces structural defects by gas phase reactions during thermal processing. A few papers [5,6,7,8,9] studied the diffusion of fluorine, germanium and other dopants during preparation of doped silica preforms and fiber drawing. High temperature and long process time cause flattening or broadening of dopant profiles.

Diffusion effects in single rod or preform processing (e.g., modified chemical vapor deposition (MCVD), outside vapor deposition (OVD), sintering after solution doping, cane stretching) are typically associated with dopant material transfer from preform surface to environmental atmosphere. The surface concentration of dopants tends to a lower value at sufficient inert gas purging. An example is the dip in the germanium doped core in typical multimode or single mode fibers in absence of additional etching procedures during preform collapsing [10].

During the fabrication of an all-solid MOF, the initial preform structure with its interstitial volumes and single stacking elements transforms into an all-solid interface structure. Using doped silica materials the simultaneous sintering and stretching process is strongly accompanied and influenced by diffusion and gas phase reactions of dopants. In this paper we simulate the dopant profile changes and gas bubble formation in germanium and fluorine doped packages of all-solid MOF preforms during cane and fiber drawing and give a short verification with drawn cane and fibers.

## 2. Thermodynamical, Kinetic and Geometrical Approximations

For exact adjustment of a stack of several elements that are prepared to draw the fiber or cane, three main effects have to be generally considered:

(1) Thermal depletion (“dilution”) of dopant due to chemical reactions and evaporation processes in the preform (*i.e.*, gas emission reactions);
(2) Change of the dopant concentration profile due to diffusion during multiple thermal processing (MCVD sintering and collapsing, core rod stretching, cane drawing, fiber drawing);
(3) Geometrical mismatch by sintering of the interstitial volume of the hexagonal package arrangement.

In the following, these three points will be addressed in detail. The presented concept finally gives a practical estimation.

### 2.1. Gas Emission Reactions

Different silica dopants, e.g., germanium, fluorine, boron, and phosphorus show evaporation effects during thermal process steps.

Germanium doped silica shows gaseous emissions according to the following simplified reaction:

GeO_2_ ↔ GeO (g)↑ + ½O_2_↑ (1)

Fluorine dopants deplete in the glass matrix according to the reaction [11]:

4SiO_1.5_F ↔ SiF_4_ (g)↑ + 3SiO_2_ (2)

For demonstration the equilibrium partial pressure is calculated for a starting dopant concentration of 10 mol% GeO_2_ (reaction in Equation (1)) and 10 mol% SiO_1.5_F (reaction in Equation (2)) in a SiO_2_ matrix, using the chemical equilibrium calculation program HSC [12]. The calculation assumes a standard pressure of 1 bar, and a dopant concentration at the surface that is not influenced by diffusion.

**Figure 1:**
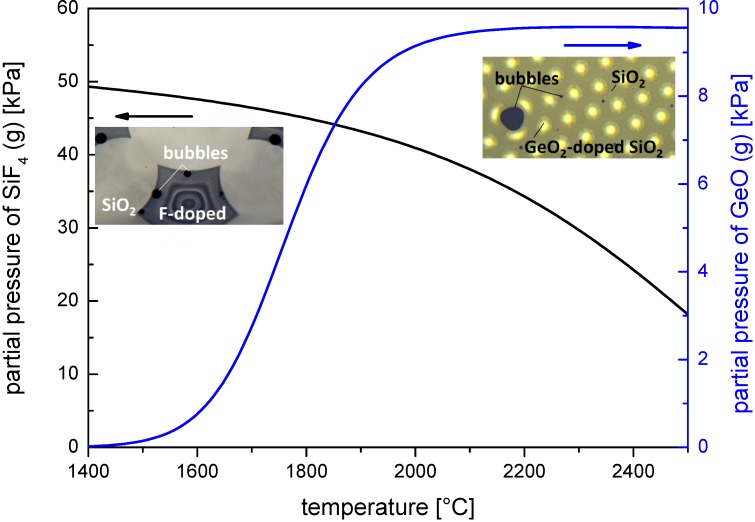
Simulated partial pressure of reaction products according to reactions in Equations (1) and (2) as a function of temperature. Black curve: SiF_4_; and blue curve: GeO. The inset micrographs exemplarily show the formation of gaseous products during fiber drawing at the contact area between doped und undoped stack elements (black areas inside fiber structure).

Figure 1 shows the simulated partial pressure as a function of temperature for evaporation reactions of fluorine and germania solved in a silica matrix according to reactions in Equations (1) and (2). The graph visualizes the strong evaporation tendency for fluorine and germanium dopants at typical fiber drawing temperatures around 1900 °C: the partial pressure amounts to about 40 kPa for SiF_4_ and to about 6 kPa for GeO.

Gaseous emissions according to Equations (1) and (2) take place as soon as the dopants are enriched near the surface (see insets Figure 1). However, in the case of GeO_2_-doped elements, the GeO_2_-doped region is usually the core surrounded by a much thicker SiO_2_ cladding; a reaction according to Equation (1) is thus not expected. Opposite is the case for SiF_4_-doped elements that are usually tubes that consist of uniformly doped SiO_2_. Thus, the SiO_1.5_F can freely access the surface and thus thermal degradation reaction according to Equation (2) can take place.

### 2.2. Diffusion Approximation

#### 2.2.1. General Diffusion Considerations

In addition to thermally induced effects of dopant depletion, there also appear kinetic limitations due to dopant diffusion. Dopant diffusion has been evaluated during MCVD processing of silica preforms with various dopants [5,6,8,13]. The basic concepts developed there will be applied to the fiber drawing process. This will yield a rough estimate of dopant diffusion effects encountered during drawing process.

Diffusion effects of dopants during fiber drawing or cane stretching broaden the concentration profile. There is an interaction between the thermodynamic effects of gas emission reactions and kinetic effects of diffusion processes. The dopant transport from the inner of the preform or cane to its surface is determined by the evaporation rate and the volume of dopants, and it also impacts the above described (thermal) interface reaction. The diffusion process is modeled according to Fick’s second law in one-dimensional coordinates:

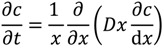
(3)
where *c* is the local concentration of the dopant, *t* is the time, *x* is the coordinate and correlates to the radius, and *D* is the diffusion coefficient.

The concentration profile is obtained from solving numerically the integral of Equation (3) where a possible solution is given by:

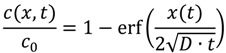
(4)

The characteristic diffusion length, *L* is obtained from the solution of diffusion in Equation (4) setting erf(1), that is *t* = τ_D_, the effective time of diffusion:

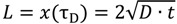
(5)

It is known that the dopant diffusion coefficients are a function of temperature *T* and of dopant concentration *c*. The temperature and concentration correlation has been investigated by Kirchhof *et al.* [6] on the basis of MCVD processing. An empirical equation (Equation (6)) has been postulated describing the observed *D* for binary SiO_2_-GeO_2_ glasses in the temperature range of 1200–2000 °C and at a concentration range from 0 mol% to 20 mol% GeO_2_. A similar dependency was obtained for binary SiO_2_-SiF_4_ glasses over the temperature range of 1000–2000 °C and for concentrations of <1 mol% of SiF_4_:

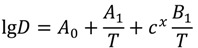
(6)
where *A*_0_, *A*_1_, *B*_1_, *x* are experimentally estimated parameters listed in Table 1. This equation is similar to the common Arrhenius expression of the (concentration dependent) diffusion coefficient:

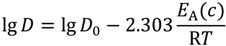
(7)
where *E*_A_(*c*) is the concentration dependent activation energy, R is the universal gas constant, *D*_0_ is the pre-exponential factor, and *T* the temperature.

The activation energies *E*_A_ can be deduced from comparison of Equations (6) and (7) using the parameters given in Table 1 as:

*E*_A_ = −2.303R(*A*_1_ + *c^x^B*_1_) (8a)

lg*D*_0_ = *A*_0_ (8b)

**Table 1:** Experimentally estimated parameters for temperature and concentration dependent diffusion equation (Equation (6)).

Dopant	*A*_0_	*A*_1_ (K)	*B*_1_ (K)	*x*	*E*_A_ (kJ/mol)	Reference
GeO_2_	3.63	−30800	71	1.0	(590-1.4*c*)	[6]
SiF_4_	0.24	−20000	0	0	383	[5]

Note that the fluorine diffusion is assumed to be independent of concentration in a concentration regime up to 6 mol% of SiF_4_.

For simulation of germanium and fluorine concentration profiles due to dopant diffusion during the drawing process, the axial length of diffusion zone in the drawing furnace is estimated in analogy to the method described in reference [13].

**Figure 2:**
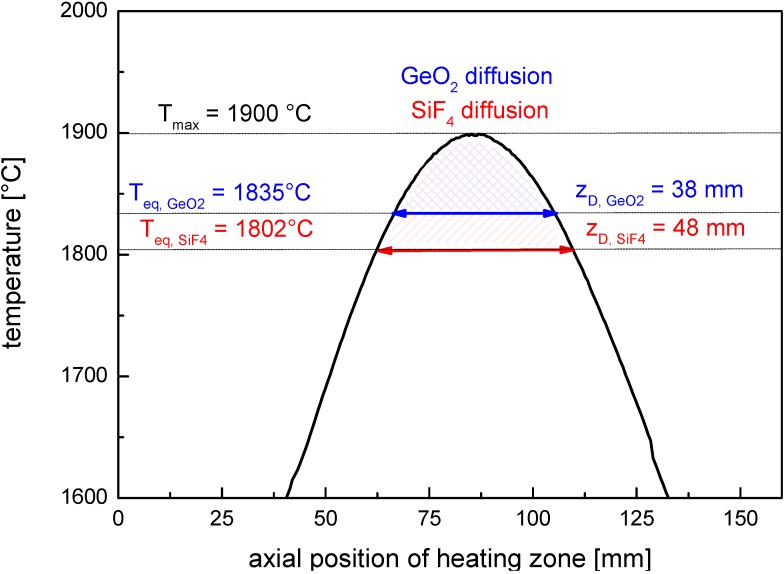
Axial temperature profile of drawing furnace as determined from thermocouple measurements with a maximum temperature of 1900 °C. The effective lengths *z*_D_ for radial diffusion of GeO_2_ and fluorine are marked in the curve in blue and red, respectively.

One important parameter for the simulation is the time the preform is exposed to such high temperatures in the furnace that noticeable diffusion of the dopants takes place. This time is governed by the speed of movement of the preform through the furnace as well as the heat zone length it is moving through. The heat zone length is the length where the temperature is sufficiently high enough for detectable diffusion. Thus, a temperature has to be estimated where the activation energy of diffusion induces a measurable dopant material flux. This temperature is called equivalent temperature *T*_eq_ and can be estimated as follows [14]:

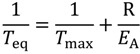
(9)
where *T*_max_ is the maximum temperature of the axial temperature profile, and *E*_A_ is the activation energy of dopant diffusion as can be calculated by Equation (8a), and R is the universal gas constant. Considering the intersection of equivalent temperature *T*_eq_ with axial position of the drawing temperature profile an effective axial heat zone length can be assessed which represents the effective length of diffusion, *z*_D_, during drawing/stretching. Figure 2 shows the estimated germanium and fluorine diffusion zone lengths *z*_D_ for a maximum temperature of 1900 °C. *z*_D_ is about 38 mm for germanium diffusion and 48 mm for fluorine diffusion, respectively. The time the preform needs to pass that distance in the furnace at a given preform speed *v*_p_ is called the effective diffusion time τ_D_. For fiber or cane drawing the preform feed rate determines the fiber drawing speed or cane drawing speed by fixed preform and fiber or cane diameters. The diffusion length *z*_D_ can be expressed either by preform or fiber material flow conditions:

*z*_D_ = *v*_p_ ∙ τ_DP_ = *v*_F_ ∙ τ_D__F_ (10)

where *v*_p_ is preform feed rate, τ_DP_ is the diffusion time on the preform scale, *v*_F_ is the fiber drawing speed, and τ_D__F_ is an equivalent diffusion time in terms of fiber scale. Equation (10) results from mass conservation during the elongation of the preform to the fiber. The effective diffusion time is needed to estimate the diffusion on a radial scale of preform or fiber.

The simulation of changes in dopant concentration profiles due to diffusion was done with COMSOL^®^ Software (COMSOL AB, Stockholm, Sweden) [15]. It is based on solving Equation (3) numerically taking into account:

- The axial temperature profile of the heating furnace;
- A step index profile of doped preform;
- Temperature and concentration dependent coefficient of diffusion which varies:
  ○ Along the relevant section of temperature profile of the heating furnace between *T*_eq_ and *T*_max_ as displayed in Figure 2;
  ○ As a function of actual dopant concentration.

The broadening of the dopant profiles can be roughly estimated from the characteristic diffusion length, *L*, which is obtained from Equation (5) setting *t* = τ_D_, the effective time of diffusion. For the fiber scale, it follows:

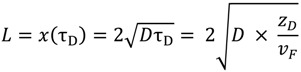
(11)

Note that *L* can be calculated either for the preform scale using τ_D,P_ or for the fiber scale using τ_D,F_ as the effective time of diffusion and substitution according to Equation (10).

#### 2.2.2. Effects of Germanium Diffusion

Germanium diffusions effects are considered for typical stacking elements that contain a GeO_2_ doped core and a SiO_2_ cladding. Thus evaporation effects according reaction in Equation (1) are negligible.

At a typical drawing temperature of 1900 °C the germanium diffusion coefficient increases by about a factor of 3 when increasing the GeO_2_ concentration from 1 mol% to 20 mol%, that is an increase from 1 × 10^−10.5^ cm^2^·s^−1^ to 1 × 10^−10^ cm^2^·s^−1^, respectively [6]. A slightly higher coefficient was estimated for fluorine diffusion. At 1900 °C, it was determined to be 1 × 10^−9.44^ cm^2^·s^−1^ [5].

**Figure 3:**
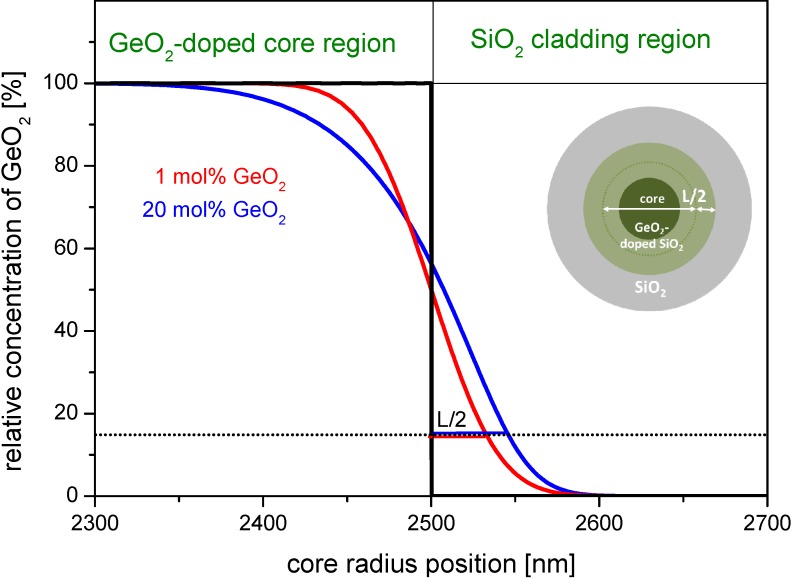
Simulated dopant concentration profiles due to diffusion in the boundary region for germanium doped step index cores with a starting diameter of 5 µm at a drawing temperature of *T*_max_ = 1900 °C and one drawing pass (simulated for fiber geometry conditions). Black curve: assumed initial profile; red curve: simulated profile starting with 1 mol% GeO_2_; and blue curve: simulated profile starting with 20 mol% GeO_2_. The inset figure shows a sketch of a GeO_2_-doped element, the dark green region represents the original concentration; and the light green represents the region with lower concentration.

Figure 3 exemplarily shows a simulated GeO_2_-concentration curve as a function of radial position. It simulates the diffusion of GeO_2_ from the center of the core towards the cladding. The initial concentration profile is a step index profile of 5 µm diameter. The effective diffusion time τ_D_ used for the simulation is evaluated from Equation (10) using a drawing speed of *v*_F_ = 10 m·min^−1^ and the afore determined *z*_D_. The regions of SiO_2_ cladding and GeO_2_-doped core are marked in the upper part of the figure.

The dopant profiles of the drawn fiber broaden by *L*/2, that is about 32 nm for 1 mol% GeO_2_ and by 45 nm in case of 20 mol% GeO_2_ doping. The relative error of this broadening at the very high doping concentration of 20 mol% GeO_2_ amounts to maximum 6% for a typical core size of 5 µm, and is significantly less for lower dopant concentrations.

Practically spoken, the penetration of GeO_2_ into the surface area of the undoped silica cladding can be neglected for most drawing scenarios.

#### 2.2.3. Effects of Fluorine Diffusion

A different situation is faced during drawing of active doped filament canes and fibers which are overcladded with a highly fluorine doped silica jacketing tube. Here, the SiF_4_-doped SiO_2_ region is directly adjoined by air. Thus evaporation reactions according to reaction in Equation (2) have to be taken into account.

Fluorine doped silica tubes and rods (e.g., Heraeus Fluosil^®^ from Heraeus Quarzglas GmbH & Co. KG, Hanau, Germany) are used for low index overcladding of laser fiber pump cores and/or the introduction of non-guiding/symmetry-breaking elements to increase the pump efficiency. The typical fabrication approach is the arrangement of rods in a fluorine doped overcladding tube.

The expected change in SiF_4_ concentration profile was again simulated with COMSOL^®^ Software [15]. Due to the high vapor pressure at the SiO_2_-SiF_4_ equilibrium (see Figure 1) under drawing conditions, the fluorine dopants directly leak into the space between preform stack and cladding. Thus, a zero concentration of fluorine at the surface during diffusion is assumed for the simulation.

**Figure 4:**
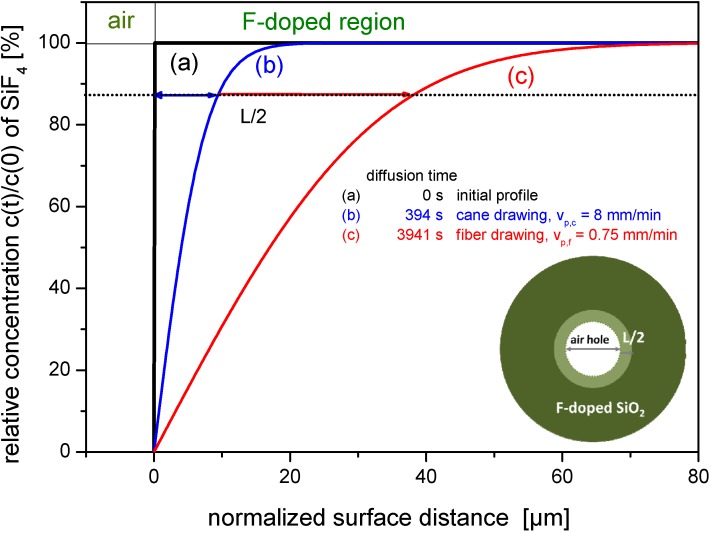
Simulated profiles of fluorine diffusion as a function of time starting with a step index profile (simulated for preform geometry conditions): (**a**) assumed initial profile; (**b**) simulated profile after 394 s; and (**c**) simulated profile after 3941 s. The inset figure shows a sketch of an F-doped tube, the dark green region represents the original F-concentration, the light green represents the region with lower concentration.

Figure 4 shows the simulated progress of the fluorine dopant profiles starting with the initial step index profile (Line a) and for the cases of cane drawing and fiber drawing (Lines b and c, respectively).

Exemplary values of diffusion lengths are given for a fluorinated overcladding tube with a mole fraction of *x*_SiF4_ = 5.4 mol%, an outer diameter of 25 mm and an inner diameter of 19 mm, thus *d*_0_ = 3 mm; at *T*_max_ = 1900 °C, a diffusion length of *L* = 9 µm for cane drawing (*v*_P_ = 8 mm·min^−1^) can be estimated, whereas for fiber drawing the equivalent diffusion length is enlarged to *L* = 35 µm due to the longer process time with *v*_P_ = 0.75 m·min^−1^.

The surface transfer of fluorine dopants into the space between preform stack and cladding creates SiF_4_-rich bubbles during drawing process. In the following, we approximate the expected volume of SiF_4_ bubbles surrounding a silica rod stack with a fluorinated silica tube.

The assumption is that all of the diffused SiF_4_ is completely evaporated into the cavities. The corresponding molar amount of vaporized SiF_4_ is converted into an equivalent bubble volume using the ideal gas equation. The ratio of SiF_4_ bubble volume to the SiO_2_ solid volume can then be expressed in the simplified form:

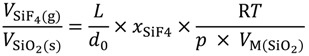
(12)
where *V*_SiF4(g)_ is the volume of evaporated gas SiF_4_, *V*_SiO2(s)_ is the volume of the overcladding tube, *L* is the characteristic diffusion length of fluorine, *d*_0_ is the wall thickness of the fluorinated cladding tube, *x*_SiF4_ is the mole fraction, and *V*_M(SiO2)_ is the molar volume of the matrix material SiO_2_. For simplification, standard conditions for the gas phase (*T* = 273 K, and *p* = 101325 Pa) are assumed.

Using Equation (12), and inserting *x*_SiF4_ = 5.4 mol% and *d*_0_ = 3 mm, a bubble volume of 11% and 35% relative to the overcladding material volume for the drawn cane and fiber can be estimated, respectively. Note that the difference in those values results from the different times the preforms stays in the heat zone of the oven: at high fiber drawing speed the preform motion is slow, whereas in case of cane drawing the preform motion is higher.

### 2.3. Geometrical Approximation

Besides thermodynamical and kinetic effects during the drawing process that might impact the geometry of the final fiber or cane, a basic geometrical approximation has to be taken into account.

**Figure 5:**
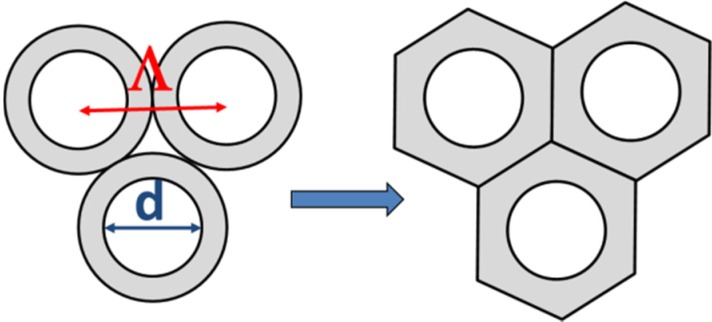
Schematical drawing of geometrical conditions (**left**) before and (**right**) after drawing.

While the elements have a circular cross section, during the sintering process in the hot zone of the oven a hexagonal shaping occurs. Since mass flow conditions always hold true, the cross sectional area of the initially circular rods is transferred to that of hexagonal elements. So the distance of the centers is slightly distorted. Such a shift of the ratio *d*/Λ is caused by the disappearance of the interstitial volume during drawing. An illustration is given in Figure 5.

From geometrical consideration, the shift can be described by Equation (13):

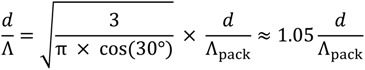
(13)
where Λ_pack_ is the distance of the core centers in the starting package arrangement. Λ_pack_ corresponds to the outer diameter of the packing rods. This demonstrates that a shift of *d*/Λ of +5% is expected.

## 3. Fiber and Cane Drawing Experiments

In this section, qualitative proof to the estimations is given exemplarily on the basis of previous drawing experiments from our lab.

### 3.1. GeO_2_-Doped Fiber

**Figure 6:**
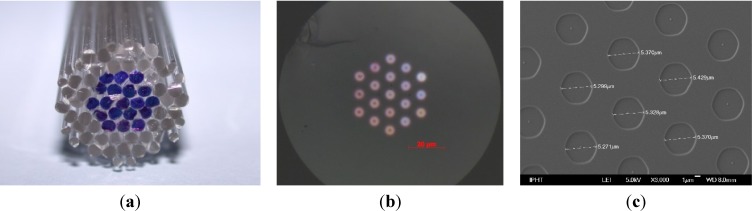
(**a**) Rod arrangement of the 19 core fiber: the GeO_2_ doped rods are marked blue; (**b**) cross sectional view of fiber after drawing; and (**c**) scanning electron microscope (SEM) image.

Filamented fibers with 19 germanium doped cores (core diameter: *d* = 5 µm, and pitch: Λ = 10 µm) were manufactured by stack-and-draw technique for preparation of a spectroscopic probe (see Figure 6) [16].

The preform package was prepared with undoped and germanium doped silica rods of outer diameter of each 1000 µm as displayed in Figure 6a. The 19 germanium doped rods with outer diameter 1000 µm and core diameter of 504 µm were manufactured by drawing from preform (preform diameter: 9.82 mm, and Ge doped core diameter: 4.97 mm). The dopant mole fraction was 6 mol% GeO_2_ in a step index profile, prepared by MCVD with 25 equivalent layers. During the stretching of the preform to the 1 mm rods the core-clad-ration *d*/Λ = 0.504 remains constant, *i.e.*, no significant broadening caused by GeO_2_ diffusion was observed. However, after drawing the final 19 core fiber from the hexagonal package (Figure 6b,c) analysis of cross-sectional scanning electron microscope (SEM) images revealed a ratio *d*/Λ ~ 0.532–0.533 for the 19 cores (GeO_2_ core diameter of all 19 elements: 5.31–5.57 µm, and pitch: 9.98–10.45 µm). This result exactly corresponds to the *d*/Λ shift as predicted by Equation (13). It is thus concluded that the *d*/*Λ* shift is mainly affected by the diminishment of interstitial volume; and insignificantly by GeO_2_ diffusion as was predicted in Section 2.2.2.

### 3.2. F-Doped Fiber

A multiple hexagonal rod arrangement of 271 different active and passive doped silica rods was surrounded with a fluorinated tube of outer diameter 25 mm, inner diameter 19 mm and a fluorine mole fraction of 5.4 mol% SiF_4_. The stacked preform was drawn to a cane of a 3 mm diameter and followed by a second drawing step into a UV acrylate coated fiber of 250 µm diameter.

**Figure 7:**
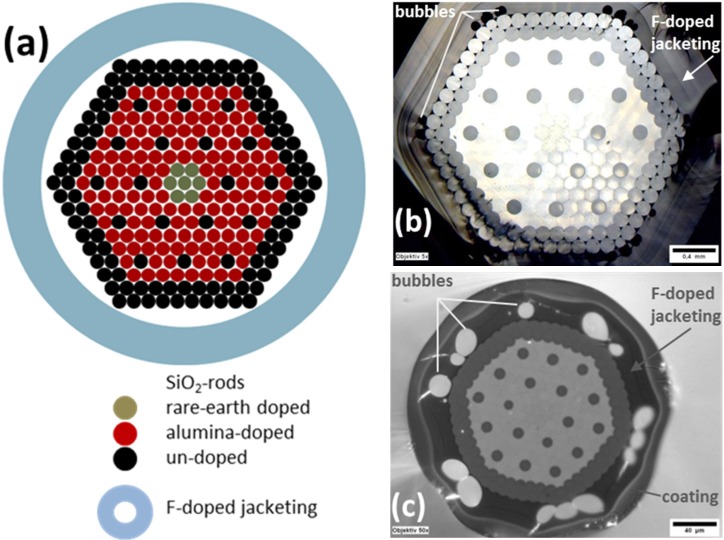
(**a**) Stacking plan of doped and undoped rods in a fluorinated tube: the white spots represent voids; (**b**) cross-sectional image of the drawn cane of a diameter of 3 mm; and (**c**) cross-sectional image of the drawn fiber of a diameter of 250 µm. Both (**b**) cane and (**c**) fiber were drawn from the same preform (**a**) prepared by overcladding the package with a high fluorine doped tube.

Figure 7 exemplarily shows the micrographs of cross sectional views of the cane (Figure 7b), and of the final fiber (Figure 7c). The bubble volume was exemplarily determined from those microscopic images by analyzing the area of the bubbles and the cross sectional area, and assuming that the bubbles remain constant over the length. In case of the cane (Figure 7b), a bubble fraction of 11 vol% was determined; and in case of the fiber (Figure 7c) a bubble fraction of 35 vol% was determined. The observed relative bubble volume is much higher in the case of the fiber than in case of the cane. The result corresponds to the approximation detailed in Section 2.2.3.

In the images of Figure 7b,c, the hexagonal shape of the cross section of the cane arises from collapse of outer tube onto the stacked elements. The formation of bubbles at the interface between stack and overcladding has been observed. Due to the drawing conditions, *i.e.*, temperature and applied drawing force, the bubbles tend to agglomerate near the “edges” of the stack and tend to get deformed (drawing force *versus* surface tension). Because of the very high fiber drawing speed compared to the much lower cane drawing speed, the created bubbles are practically frozen (in a non-spherical shape) in the fiber. The viscosity-driven “bubble freezing process” is too fast as to allow for compensating inner pressure and surface tension effects. Thus, the formed bubbles are more likely to remain deformed in the fiber as compared to the cane drawing process.

## 4. Conclusions

This paper described diffusion based manufacturing aspects of all-solid MOFs relating to volatile dopants. Geometrical considerations have been included. The main conclusions are that under typical fiber drawing conditions:

(1) The effect of dopant diffusion is marginal for typical preform dimensions: dopant diffusion lengths in the preform have been approximated to be about 5 µm for typical GeO_2_-doped stacking elements, and 30 µm for typical SiF_4_-doped material.
(2) Formation of bubbles in the preform, cane and fiber might occur due to outgassing of thermal degradation products of the dopants. Such gaseous emissions take place as soon as the dopants are enriched near the surface.
(3) A geometrical mismatch of +5% has to be taken into account for the *d*/Λ ratio due to the sintering of stacking elements and disappearing interstitial volume.

The chemical equilibrium conditions for surface evaporation reactions at typical drawing or stretching temperatures of doped silica cause a relative high partial pressure of some gaseous products. The approximation of evaporation reactions at 1900 °C shows a partial pressure for germanium monoxide of about 0.1 bar, for silicon tetrafluoride of about 0.45 bar with an exemplary dopant level of 10 mol% GeO_2_ or SiF_4_, respectively. The various bubble sizes—observed during cane and fiber drawing of highly fluorinated preform components—are caused obviously by the diffusion control of the SiF_4_ evaporation. As a consequence inserting a sufficiently thick barrier layer (thickness larger than diffusion length *L*) would be capable of efficiently suppressing unfavored evaporation effects.

Diffusion effects encountered during the drawing process lead to a broadening of the dopant profile. This is caused by the typically large heating zone and long dwell time in industrial drawing furnaces, configured for the processing of large preforms. The GeO_2_ dopant profile broadening seems to be insignificant for fiber core cross sections of >5 µm, e.g., typical single mode fibers (Ø 5–10 µm) or large core fibers (*i.e.*, large mode area laser fibers, multi-mode fibers). Exemplarily, the simulation predicts for a fiber core of 5 µm diameter and a low germanium concentration of 1 mol% GeO_2_ a relative broadening of the core diameter of about 1% in one thermal drawing pass. It approximately doubles for high GeO_2_ concentration of 20 mol% GeO_2_. At much smaller core sizes or doped layer thicknesses where the dimensions are comparable to the diffusion length the free choice of smart geometrical designs of MOFs is limited. So for typical preform cross sections and fiber drawing conditions, we expect in case of very small doped cores with *d* ≤ 1 µm, e.g., fibers for non-linear applications, a relative increase of the core diameter of ~10%–20% compared to the preform parameters. To overcome this limit, micro-drawing techniques with small sized preforms have to be applied.
